# Supplementary material for: Intelligent Liver Function Testing (iLFT): An Intelligent Laboratory Approach to Identifying Chronic Liver Disease
Source: Diagnostics (Basel). 2024 May 4;14(9):960. doi: 10.3390/diagnostics14090960 (PMC11083526; doi:10.3390/diagnostics14090960)
Supplement: Supplementary file 1 [file diagnostics-14-00960-s001.zip › diagnostics-2984340-supplementary.pdf]

Supplementary material: intelligent Liver Function Testing (iLFT): an intelligent laboratory approach to identifying chronic liver disease

Software

Order communications software: Sunquest ICE v7.1.5 – Sunquest Information Systems Inc.
Laboratory information management system (LIMS): CliniSys LabCentre v1.14 – CliniSys Solutions Ltd.
Laboratory Data Management Software: Atellica Data Manager v1.3.1.13 – Siemens Healthcare Diagnostics Inc. [Previously, CentraLink® Data Management System – Siemens Healthcare Diagnostics Inc.]
Middleware: Instrument Manager v8.17.31.03 – Data Innovations LLC

Automation

Aptio Automation technology – Siemens Healthcare Diagnostics Inc.

Analytical platforms and methods

Biochemistry

All biochemical assays are currently performed on the Atellica Solution Clinical Chemistry & Immunoassay analyzers, with the exception of ceruloplasmin and alpha-1 antitrypsin phenotyping which are referred to external laboratories.

Individual methods are detailed below (all Siemens Healthcare Diagnostics Inc.):

Alanine aminotransferase: Atellica CH Alanine Aminotransferase P5P (ALTPLc)
Albumin: Atellica CH Albumin BCP (AlbP)
Alkaline phosphatase: Atellica CH Alkaline Phosphatase, Concentrated (ALP_2c)
Alpha-1 antitrypsin: Atellica CH Alpha-1-antitrypsin (AAT)
Aspartate aminotransferase: Atellica CH Aspartate Aminotransferase (AST)
Bilirubin: Atellica CH Total Bilirubin_2 (TBIL_2)
C-reactive protein (CRP): Atellica CH Wide Range C-Reactive Protein (wrCRP)
Direct (conjugated) bilirubin: Atellica CH Direct Bilirubin_2 (DBIL_2)
Gamma-glutamyl transferase: Atellica CH Gamma-Glutamyl Transferase (GGT)
Haptoglobin: Atellica CH Haptoglobin (Hapt)
Hepatitis B surface Antigen: Atellica IM Hepatitis B surface Antigen II (HBsII)
Hepatitis C IgG Antibodies: Atellica IM Hepatitis C (aHCV)
Hyaluronic acid: Atellica IM Hyaluronic Acid (HA)
Iron: Atellica CH Iron_2 (Iron_2)
N-terminal propeptide of type III procollagen (PIIINP): Atellica IM N-terminal Propeptide of Type III Procollagen (PIIINP)
Transferrin: Atellica CH Transferrin (Trf)

Tissue inhibitor of matrix metalloproteinase-1 (TIMP-1): Atellica IM Tissue Inhibitor of Matrix Metalloproteinase-1 (TIMP-1)

Calculated tests: Percentage saturation of transferrin, Enhanced liver fibrosis score (ELF), Fibrosis-4 index (FIB-4), NAFLD fibrosis score (NFS)

[Previous platforms: ADVIA 2400 Clinical Chemistry System [ALT, ALP, total bilirubin, albumin, GGT, iron, CRP], Dimension Vista 1500 System (alpha-1 antitrypsin, AST, direct bilirubin, ferritin, haptoglobin), ADVIA Centaur XP Immunoassay System (TIMP-1/PIIINP/HA (for ELF), hepatitis B surface antigen, hepatitis C IgG antibody) – all Siemens Healthcare Diagnostics Inc.]

Haematology

Platelet count: Laser light scatter, ADVIA® 2120i Hematology System – Siemens Healthcare Diagnostics Inc.

Immunology

Liver antibody screen: Indirect immunofluorescence on rat triple block tissue using QUANTALyser 2 – Inova Diagnostics Inc.

Anti-nuclear antibody (ANA): Indirect immunofluorescence on HEp-2 slides using QUANTALyser 2 – Inova Diagnostics Inc.
